# Digital Twin for Volumetric Thermal Error Compensation of Large Machine Tools

**DOI:** 10.3390/s24196196

**Published:** 2024-09-25

**Authors:** Beñat Iñigo, Natalia Colinas-Armijo, Luis Norberto López de Lacalle, Gorka Aguirre

**Affiliations:** 1 Design and Precision Engineering, IDEKO, 20870 Elgoibar, Spaingaguirre@ideko.es (G.A.); 2 Department of Mechanical Engineering, University of the Basque Country (UPV/EHU), 48013 Bilbao, Spain

**Keywords:** digital twin, machine tool, calibration, volumetric error, thermal compensation

## Abstract

Machine tool accuracy is greatly influenced by geometric and thermal errors that cause positioning deviations within its working volume. Conventionally, these two error sources are treated separately, with distinct procedures employed for their characterization and correction. This research proposes a unified volumetric error compensation approach in terms of a calibration procedure and error compensation model, which considers geometric and thermal errors as a single error source that exhibits temporal variation primarily due to changes in the machine’s thermal state. Building upon previous works that introduced a fully automated volumetric calibration procedure capable of characterizing the variation in volumetric error over time, this study extends this methodology, incorporating multiple temperature sensors distributed throughout the machine and generating a digital twin based on a volumetric error compensation model capable of predicting and compensating for the volumetric error over time at any point in the workspace, using temperature measurements and axis positions as inputs. This methodology is applied to a medium-sized milling machine tool. The digital twin is trained and validated on volumetric calibration tests, wherein various controlled heat sources are employed to induce thermal variations while measuring the temperatures in the machine.

## 1. Introduction

Machine tool accuracy is of the utmost importance in modern manufacturing industries, where even small errors can significantly affect the dimensional accuracy of manufactured parts. Geometric and thermal errors are among the primary error sources regarding the volumetric accuracy of machine tools and have been of major concern for decades [1,2,3,4]. Historically and up to the present, these two error sources have been treated separately, as, for example, in [5,6], with distinct procedures employed for their characterization [7,8].

The characterization of and compensation for the geometric errors affecting the volumetric accuracy of machine tools have been widely discussed, as reviewed in [6,9,10]. State-of-the-art technology for characterizing the geometric errors in medium- and large-sized machine tools often involves the use of Laser Trackers (LT) and multilateration-based solutions. The standard solution is to position a Laser Tracker on the ground or the table at different locations to measure a machine’s geometric errors [11,12,13], while another approach involves attaching a tracker to the machine head and using fixed reflectors on the ground [14]. An alternative, cost-effective method utilizes artifact-based solutions. Although these may have limitations in terms of range and measurable positions, the cost of the equipment can be an order of magnitude below that of the previous methods, while maintaining a similar precision. Early techniques for volumetric error mapping involved measuring a calibrated artifact or standard at various positions within a machine’s workspace [15], and different procedures have been developed since [16,17,18]. Comprehensive reviews of artifact-based machine error mappings can be found in the literature [19,20]. These error-mapping solutions primarily focus on the geometric errors of machines, which are mainly caused by manufacturing errors in the guideways, assembly errors, and the flexibility of structural elements [21,22,23].

In the context of prior research, approaches to handling the effects of thermal errors in volumetric calibration can be broadly divided into two categories. The first category of approach assumes or requires that thermal conditions remain stable and have a minimal impact on the characterization of geometric errors, as in [24,25] and most ISO standards regarding geometric errors [26,27], where stable thermal conditions or previous warmup cycles are recommended. However, this assumption is often impractical, particularly in industrial settings and large machines, where maintaining stable thermal conditions becomes challenging. The second category of approach is more reliable, as it incorporates thermal effects as an uncertainty source, which influences calibration accuracy [17,28,29,30]. However, there is not a unified methodology for assessing thermal uncertainties, and these approaches often rely on simplifications, such as considering only offset drifts and linear expansion or assuming uniform and linear temperature changes. These oversimplifications may lead to either an overestimation or underestimation of the actual thermal effects. Thus, a more comprehensive and systematic evaluation of thermal influences is essential for evaluating the precision and performance of calibration procedures.

In fact, the significance of managing thermally induced errors in machine tools is being increasingly recognized by academia and the manufacturing industry. International standards have been developed to assess thermal behavior [31,32], with updates as recent as 2022 [33], which now include standard machining tests to evaluate thermal distortion. Advances in measurement equipment, mechatronics, and computational techniques have led to better estimations of temperature distribution and thermally induced displacements at the Tool Center Point (TCP) [34]. As the manufacturing industry seeks a higher precision for machine tools, the predictability of thermal stability becomes critical in order to avoid costly design modifications later in machine development. The efforts to reduce such thermal effects in the early stages of machine conception can be broadly divided into trying to minimize the thermal distortion either by design [35,36,37,38] or by assuring thermal stability with cooling or thermal shields [39].

On the other hand, thermal error compensation by the means of digital twins can be a cost-effective solution to reduce the influence of thermal errors in machine precision, and can complement previous thermal error reduction strategies, even when the machine is already in production. Comprehensive reviews of thermal compensation strategies can be found in the literature [5,40]. The most used approach consists of training digital twins based on phenomenological models with experimental data, where the relation between inputs (e.g., temperature and power consumption) and the Tool Center Point is estimated. These digital twins are then implemented in the control of the machine, reading temperature probes and axis positions to continuously estimate the thermal error and compensate for it. These approaches include different variations of Multiple Linear Regression (MLR) [41], identifications of linear time-invariant systems like autoregressive models with exogenous inputs (ARX) [42], transfer functions [43,44], and neural networks [7,45]. In general, these works focus on measuring and predicting the thermal errors of a single machine position and lack the spatial or volumetric component of thermal behavior. This becomes especially important in big machines where the working volume can be of several meters in each direction and controlling the temperature of the whole machine frame becomes difficult.

Digital twins obtained from simulation data can, in principle, overcome this volumetric limitation. A Finite Element model of the machine is normally used, and the full deformation field can be estimated using thermal loads [46] or multiple temperature measurements [47,48] as an input. The most advanced models have the capability to add relative movements between bodies, i.e., machine axes, and, thus, reproduce the errors in the volume [49]. However, these approaches do not usually allow for model adjustments and rely on the accuracy of the simulation model, although recent efforts have been made in trying to combine both approaches [50]. The modeling of the boundary conditions in thermomechanical systems is much more complex than its mechanical counterpart, and great efforts are made to model even a single parameter or phenomenon accurately [51,52]. Such modeling efforts can make this simulation-based digital twin approach unfeasible, especially in industrial environments, where different types of machines and environments would need to be modeled while meeting demanding production deadlines.

Hence, conducting experimental training for a digital twin based on a phenomenological model appears to be a promising approach. However, certain constraints need to be addressed to ensure comprehensive error characterization across the entire volume. One of the early works capturing the volumetric behavior of thermal errors can be found in [53], where the position-dependent behavior of geometric errors is combined with a temperature-dependent first-order term. Later efforts include the temperature- and position-dependent behavior of a single axis based on measured temperature integration [54], multilateration-based full volumetric calibrations at different constant ambient temperatures [55], and the use of Position Sensitive Detectors (PSDs) to measure variations in straightness, squareness, and roll error motions [56].

In previous works, an artifact-based volumetric calibration method was proposed and optimized [57]. The procedure was implemented in a fully automated way so that it could be repeated over time without any human intervention. In [58], it was shown that this calibration procedure could be used to characterize the variation in volumetric errors over time in a medium-sized milling machine. Different thermal error sources were applied to the machine over a week-long test and different thermal behaviors were measured and analyzed. This paper took the next logical step by introducing a digital twin to compensate for the thermal errors within the machine volume. This was based on an integrated methodology that treats geometric and thermal errors as a unified error source, acknowledging their time-dependent nature, primarily driven by fluctuations in machines’ thermal conditions. The above-mentioned volumetric calibration method already extends geometric error calibration to thermal effects by repeated measurements that capture variations with time, and temperature sensors distributed on the machine structure are added to capture the variations in its temperature field. The compensation model introduced here extends an existing phenomenological volumetric error calibration model to capture the dependance of these errors with the temperature field in a machine.

A digital twin of the thermal–elastic behavior of a machine was obtained by identifying the parameters of the volumetric thermal error model from experimental volumetric calibration data, which include temperature and volumetric error data. This digital twin can be deployed in the control of the machine to improve its accuracy by continuously estimating the positioning error of the machine as a function of the axis positions, conducting temperature measurements, and applying the corresponding corrections to the commanded position of each axis.

This methodology was applied on a moving column milling machine. The research involved conducting two distinct thermal tests, each spanning multiple days. The first test, referred to as the training test, was undertaken to establish the compensation model, while the second, known as the validation test, aimed to assess the quality and reliability of the model. To comprehensively capture the machine’s thermal state, a network of 50 temperature sensors was strategically installed.

The structure of this paper is as follows: Section 2 provides a comprehensive account of the methodology employed, showing both the experimental setup and the theoretical basis of the digital twin for the volumetric thermal error compensation. Section 3 presents a summary of the experimental results. Section 4 discusses the effectiveness and reliability of the digital twin. Section 5 closes with conclusions and outlook.

## 2. Materials and Methods

### 2.1. Experimental Setup

**Figure 1:**
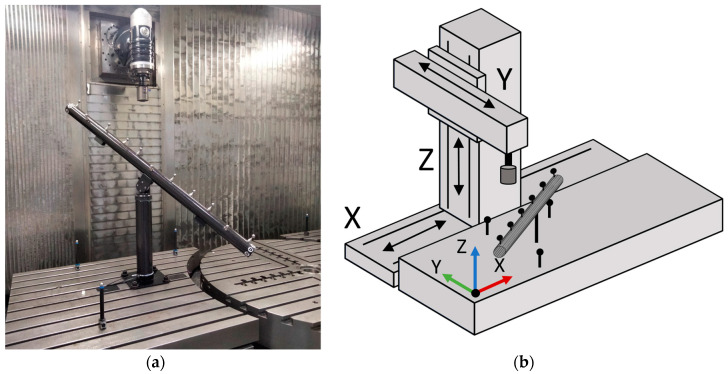
(**a**) Calibration setup with the ball array, 3 individual spheres on the machine table, and the measuring probe mounted in the machine head. (**b**) Schematic depiction of the machine kinematics. XYZ linear axes of the machine are shown with arrows indicating travelling directions. The origin and the axes of the machine reference system are shown, located on a working table corner.

Following methodology developed in previous works, an artifact-based calibration procedure is carried out in a medium-sized milling machine. It is a moving column-type milling machine, with a fixed table on the workpiece side. The actual setup is shown in Figure 1a, while a schematic view of the machine and the artifact set-up can be seen in Figure 1b.

**Figure 2:**
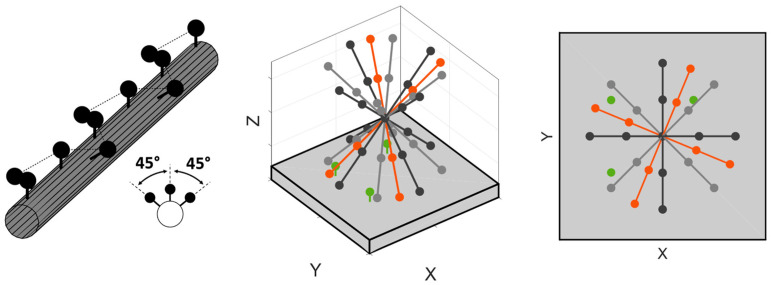
(**Left**) Design of ball array and (**center**, **right**) fixed balls and artifact orientations. Fixed balls are shown in green. Artifact positions used to obtain the model are shown in grey and black. The artifact positions used to validate the model are shown in red.

As stated in [57], the calibration process consists of measuring a ball array with high-precision spheres over several orientations inside the machine working volume. These measurements are repeated over several days in order to capture the thermal variations in the machine errors. In order to make this process automatic, the artifact is mounted in a cylindrical base with an embedded rotary motor. The inclination around the horizontal (elevation) angle is set manually and locked throughout the test, as the rotation around the vertical (azimuth) angle is provided by the rotary motor. The volumetric calibration procedure is depicted in Figure 2. Details about the calibration procedure are given in [58].

**Figure 3:**
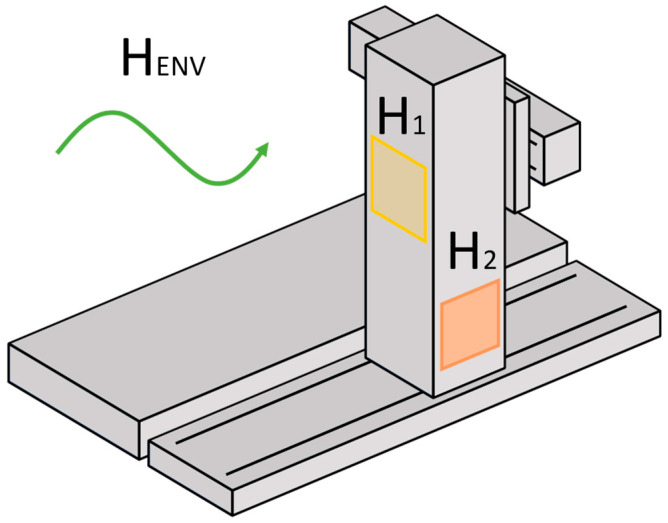
Location of the heat sources (hot air ventilators) at the back and the side of the column along with the forced ambient temperature changes.

In order to induce different thermal conditions, three controlled heat sources are implemented. Two of them are hot air ventilators, focused on the lower part of the column, one on the side (H_1_) and another on the back (H_2_). The third heat source is the climate control in the workshop (H_ENV_), which is forced to warm up or cool down the ambient temperature. Figure 3 shows a schematic view of the locations of these heat sources.

**Figure 4:**
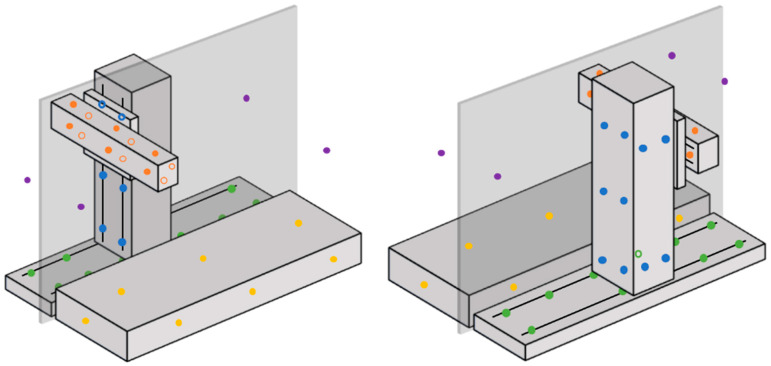
Front (**left**) and back (**right**) schematic view of the machine with color-coded temperature sensor locations: table-workpiece (yellow), X-bed (green), Z-column (blue), Y-ram (orange), and ambient (purple). Sensors hidden behind bodies are shown with blank filling.

As stated in the introduction, the ultimate goal of this work is to build a digital twin that will predict machine errors using temperature measurements as inputs. In order to map the thermal state of the machine, up to 50 temperature sensors are installed in different parts of the structure, as follows: 10 in the workpiece side table, 10 in the X axis bed, 16 in the column, 12 in the ram, and 4 ambient sensors. The working volume of the machine (the operator side) is encapsulated, and the main parts of the structure are on the back side, directly in contact with the ambient environment of the workshop. Therefore, 2 of the ambient sensors are located on the left- and right-hand sides of the working volume and the other 2 are at the back. All the temperature sensors are type-T thermocouples with an uncertainty of ±0.5 °C according to the manufacturer’s data. Figure 4 shows the approximate locations of the sensors in the machine structure.

### 2.2. Digital Twin for Thermal Error Compensation

A methodology for the generation of a digital twin that reproduces the thermal–elastic behavior of a machine is proposed here. This digital twin can be implemented in the control of such a machine to improve its accuracy by estimating and compensating for, in real time, the positioning error induced in the TCP by the thermal growth. The digital twin uses measurements from a few temperature sensors distributed within the machine and the positions of the axes of the machine as inputs. This digital twin is generated by identifying, from experimental data, the parameters of a phenomenological model that extends classical volumetric error compensation models to include the influence of temperature. This process is explained in the next three steps.

Following the procedure explained in the previous section, the volumetric error is characterized by obtaining the geometric error parameters based on the kinematic model of the machine. It consists of three linear axes, where each axis has 6 position-dependent error motions (i.e., positioning, straightness, and angular errors) along with the squareness errors between them. These errors are approximated by a series of different polynomials, whose parameters are identified in a least squares minimization procedure. More details on the obtention of these error parameters are given in [57,58]. At this point, a machine model capable of predicting the error in the volume according to the axes’ positions and the error parameters can be obtained as follows:

(1) e=M(X,E)

where X represents the axes’ positions (x, y, and z axes in this case), E is the set of parameters that define the machine motion errors, M represents the kinematic model of the machine, and e is the predicted error at the TCP. The inputs in E are considered to be parameters of the model and are represented in bold characters to differentiate them from the axes’ positions X, which are considered to be model variables. However, the model M only represents the behavior of the machine at a specific time or state. If the calibration procedure is repeated over time, the volumetric error of the machine will change, mainly due to thermal variations. In this case, different error parameters, and in consequence, different machine models, will be obtained for each time instant. Therefore, we can rewrite Equation (1) as:

(2) $$e=M^{t}(X,E^{t})$$

with the superscript t indicating the time step where the model is characterized.

With this set of machine models, the error of any point in the workspace can be predicted within the duration of the test by using the machine kinematic models and interpolating between them. However, in order to obtain a digital twin capable of predicting and compensating for error variations beyond the duration of the test, a thermal model must be obtained relating the measured temperatures and error parameters. Therefore, the previous equations can be rewritten as:

(3) $$e=M_{T}(X,E(T))$$

thus obtaining a unique digital twin of the machine capable of predicting the error in the volume and over time.

As discussed in Section 1, different thermal models have been proposed in the literature for error compensation. These approaches are usually inspired by the physics behind the thermoelastic problem, which describes the relationship between the thermal loads and the temperature field as a first-order differential equation, depending on the heat capacity, material conductivity, and other boundary conditions. On the other hand, once the full temperature field of a body is known, the deformation of any point can be instantaneously computed.

Hence, if temperature measurements are to be used as inputs for the compensation model, a direct relation should be established between them and the volumetric error, i.e., the deformation at any point should be a linear combination of the temperatures multiplied by different coefficients. Any dynamic effects derived from the differential equation problem should be left out. However, in most cases, measuring the full temperature field is unattainable, and the available information usually consists of a few scattered temperature measurements. In fact, thermal modal analysis approaches have shown that these delays can exist between individual temperature measurements and deformations [59,60,61]. On the contrary, optimal sensor positioning techniques have shown that a very accurate approximation of the temperature field can be achieved with few sensors [62,63], using direct models with no delay, if these are adequately positioned. Therefore, it is expected that choosing a model resembling partial differential equations will have benefits when using one or few temperature sensors, as models based on simple linear combinations should be used when more temperatures are available.

To answer this question, an autoregressive model with an exogenous input (ARX) is adopted. In this type of model, the evolution of a parameter is predicted according to its past values and one or several external inputs, which, in this case, refer to temperature measurements. Hence, a multiple-input single-output (MISO) model is adopted to predict each parameter. The MATLAB System Identification Toolbox is used to fit such models according to the procedure described below.

Let E be the error parameter set obtained in the calibration model, designating each individual error parameter as $E_{m}$, where $m=1,\ldots ,dim(E_{m})$, and the measured temperatures as $T_{n}$, where n=1, …, 50 (see Figure 4). Then, the ARX model predicting each parameter can be expressed as:

(4) $$E_{m}^{t}={a_{m}\cdot E}_{m}^{t-1}+\sum_{n=1}^{N}b_{n}^{m}\cdot T_{n}^{t}$$

where the parameter $a_{m}$ defines the autoregressive behavior of the parameter $E_{m}$ and the coefficients $b_{n}^{m}$ define the relationship between the $n^{th}$ temperature and the $m^{th}$ error parameter. At this point, overfitting effects must be taken into consideration in order to obtain a model as robust as possible. The so-called bias–variance dilemma [64] states that the use of excessive parameters trying to avoid model bias and minimize fitting residuals can result in a higher variance in the predictive performance outside the training set. This effect can be aggravated by the multicollinearity between temperatures, i.e., the fact that the possible inputs of the model (measured temperatures) are highly correlated and can be predictors of each other [65].

The aforementioned problems lead to the following questions:

- How many and which temperatures should be selected for each error parameter?
- Is it necessary to include the first-order autoregressive parameter $a_{m}$ in the prediction model?

For the temperature selection, an approach based on [66] is followed, called the split unbiased estimation algorithm. The temperature selection is performed in a greedy way, where, in each step, the single best input available is selected, until the final number of desired inputs is achieved. Taking Equation (4) as a basis and assuming for the clarity of the explanation that $a_{m}=0$, the procedure can be described as follows:

(1) For a specific parameter $E_{m}$, the desired number of inputs $N_{m}$ is selected. This means that, at the end of the procedure, only a limited set of $N_{m}$ temperatures will be selected out of the available N=50 temperatures.
(2) An iterative process starts with the iteration steps $j=1,\ldots ,N_{m}$. At the first iteration (j=1), a single-input single-output ARX model is fitted with each of the temperatures available, selecting the one with the lowest mean squared error between the measured and the predicted values.
  (5) $$\min\left\{E_{m}-b_{n}\cdot T_{n}\;\;\right|\;n\in N\}$$
  Naming the selected temperature as $T_{n1}$, a new output is defined for the next step of the iteration.
  (6) $$E_{m}^{*}=E_{m}-b_{n1}\cdot T_{n1}$$
(3) The procedure is repeated for the subsequent iterations, where the temperatures are selected one by one until the desired number of $E_{m}$ temperatures is reached. Naming the temperatures selected in previous steps as $N_{j}$, the fitting procedure can be summarized in the expression of Equation (7):
  (7) $$\min\left\{\;\;E_{m}^{*}-b_{n}\cdot T_{n}\;\;\right|\;\;\;n\in \left[N-N_{j}\right]\;,\;N_{j}<N_{m}\}$$

Repeating this procedure for each of the $E_{m}$ parameters, m multiple-input single-output (MISO) models are obtained, with each one predicting the behavior of individual parameters according to different temperature inputs. The predicted parameters are incorporated into the kinematic model of the machine so that the volumetric error can be estimated at any moment, thus obtaining the digital twin of the machine with real prediction capabilities for the thermal behavior in the volume.

Once the fitting procedure is defined, it is necessary to establish how many temperature inputs ($N_{m}$) are needed to predict each parameter. For this purpose, an approach based on information criterions will be used, following the methodology in [42]. Information criteria offer a quantitative approach to comparatively evaluate regression models. There exist different variations, but, in general, they prioritize a smaller squared error while discouraging the inclusion of additional parameters, thereby mitigating the risk of overfitting. The MATLAB^®^ System Identification Toolbox used in this work for the ARX model estimation already provides the values for the Akaike Information Criterion (AIC), the Bayesian Information Criterion (BIC), and the corrected Bayesian Information Criterion (BICc) for each model. The latter is used in this work.

These criterions will be used both to select the adequate number of temperature inputs and to either include or exclude the autoregression parameter $a_{m}$. Different models will be fitted for each parameter, starting with 1 temperature up to 10, with and without the autoregressive parameter. The one with the lowest BICc is selected as the best model.

**Figure 5:**
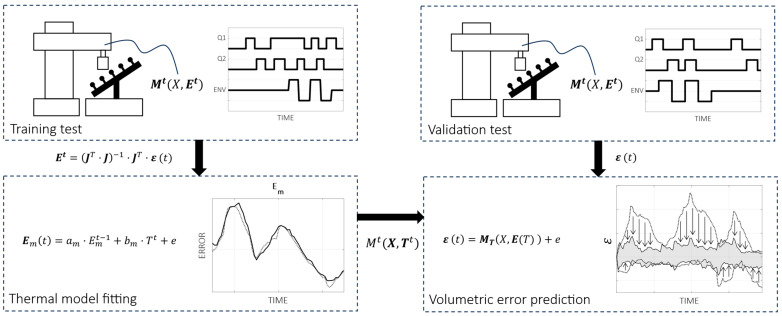
Summary of process for the obtention of a digital twin for volumetric thermal error prediction. (**Top left**) Training test with the consequent obtention of the volumetric errors changing over time; (**bottom left**) ARX regression with temperatures as inputs obtaining a compensation model; (**top right**) validation test measuring distance errors; and (**bottom right**) volumetric error prediction and compensation using model M over errors measured in validation test.

For validation purposes, two different tests are carried out, comprising the training and testing data sets, respectively. Each test is a several-days-long thermal test where the procedure explained in Section 2.1 is repeated under different thermal conditions. The model fitting described in this section will be carried out with the data from the training test. The performance of the prediction model will be evaluated with the testing data set. More details about both tests are given in Section 3. Figure 5 shows a diagram summarizing the whole process, from the experimental measurements to the error prediction and compensation.

### 2.3. Quantification and Evaluation of Thermal Errors

The accurate assessment of thermal errors is of the utmost importance when reporting the results of a thermal test or the performance of a compensation model. The typical representation of test results consists of a graph representing thermal distortion versus time, while the ISO 230—Part 3 standard [31] recommends plotting measured temperatures alongside distortions. In the case where an estimation/compensation model is involved, the estimation curve is usually included overlaying the distortion curve, and the difference between the two can be added, representing the residual error after compensation.

Additionally, different ISO standards propose machining tests to evaluate thermal distortions. These tests are based on test pieces with specific geometric features to evaluate different error components. These features are machined several times in evenly spaced time intervals and the measurements are carried out on the machine after a reasonable cooling time has passed. Section 4.4 of this work provides a simulation of the trajectories of 2 machining tests proposed by the ISO 10791—Part 10 standard [33] in Sections A.2 and A.3, respectively, where the improvement after compensation is shown, simulating the conditions of the validation test.

The numerical assessment of distortion curves typically consists of metrics such as the range (peak-to-valley value) or dispersion (RMS and standard deviation) of the measured distortion over the entire duration of the test. If a compensation model is involved, such metrics are computed before and after the compensation is applied, and the improvement rates are reported to assess the quality of the prediction model.

However, reporting thermal results in terms of single numerical values presents major limitations, as this only represents the behavior of the machine at time spans comprising the entire duration of the test. For example, a value reporting the variation in the thermal error over a 4 h long test gives little information about how the machine behaves in shorter time spans, although it provides an upper threshold. ISO standards recommend that the duration of these tests should be agreed between the manufacturer and the user of the machine, based on the typical machining times over which the machine will operate. However, a single machine will frequently work with different workpieces, operations, and referencing points with distinct machining times. Consequently, they will be affected differently depending on the changing rates of thermal distortions and the specific operation.

**Figure 6:**
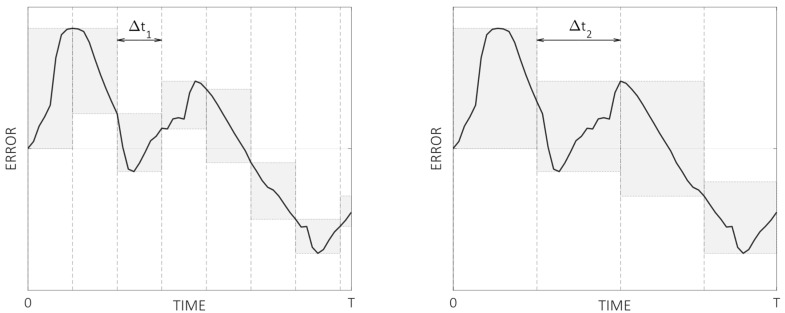
Evolution of a distortion error during a thermal test of duration T. Left and right part show the same error segmented in different time intervals $\Delta t_{1}$ and $\Delta t_{2}$. The grey areas cover the range (peak-to-valley value) of the error in a specific time interval.

To overcome this limitation, a new way to report thermal distortions is proposed in the following, which can be applied both to characterization tests or compensation models, and it is especially useful for comparing different machines, tests, or compensation strategies. Figure 6 shows the evolution of a generic thermal distortion error measured during a test of duration T. The error is segmented according to two different time intervals, $\Delta t_{1}$ and $\Delta t_{2}$ (where $\Delta t_{1}<\Delta t_{2}\ll T$), e.g., representing a hypothetical serial machining of two different workpieces, and the range of the error in each time interval is represented by the shaded area in grey. Assuming that referencing is carried out between the workpieces, it is clear that a different thermal distortion would be expected depending on the workpiece type.

At this point, different parameters can be adopted to represent the thermal distortion of each case, with some examples given in the following:

- $E_{max(range)}$: The maximum variation happens in any of the individual segments. For the example shown in Figure 6, this would correspond to the first segment for both time intervals. This would give a conservative value for the thermal behavior, representing the worst-case scenario between the measured values.
- $E_{\mu (range)}$/$E_{\sigma (range)}$: The mean value or standard deviation of the error range registered in all segments. The standard deviation can be expanded or percentiles can be used to represent different portions (e.g., $E_{P95(range)}$, meaning that 95% of time intervals registered an error below this value). This represents a statistical approach to represent the error evolution and can give a better sense of the general thermal behavior.

Other parameters can be computed with different variations, as, for example, when using the linearity error of each segment instead of a range, if it can be assumed that a constant changing rate will not affect the specific tolerance of a machining operation.

Nevertheless, the key aspect of this new approach is that each parameter should be reported, indicating the time interval with which they have been calculated. For the example shown in Figure 6, the maximum variation errors should be reported as $E_{max(range)}^{\Delta t_{1}}$ and $E_{max(range)}^{\Delta t_{2}}$ for the left and right sides, respectively, giving a sense of the machine’s thermal behavior when working at those specific time intervals.

**Figure 7:**
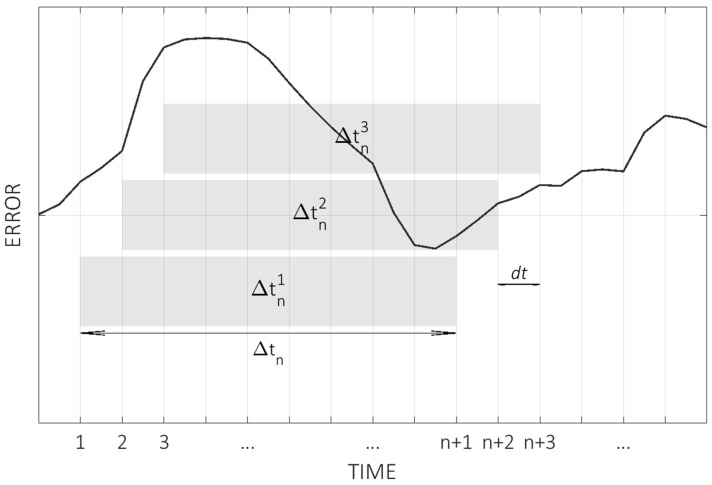
Illustrative example of sliding window sampling of time intervals of generic width $\Delta t_{n}$.

When the time intervals of interest are not defined or a more generic evaluation of thermal behavior is needed, a generalization of the previous approach can be used. Let dt be the time step between subsequent measurements, i.e., the temporal resolution of the measured thermal distortion curve. Assuming uniform sampling, time intervals can be defined starting from $\Delta t_{1}=dt$ up to $\Delta t_{N}=T$, with $N=\frac{T}{dt}$. The former represents the time interval between any pair of subsequent points, as the latter is the duration of the whole test. Instead of establishing overlayed time intervals (as used for illustrative purposes in Figure 6), intervals of the same length are taken from a sliding window of width $\Delta t_{n}$, giving a total of N−n intervals to evaluate for a time interval of $\Delta t_{n}$. This is illustrated in Figure 7.

For each of the time intervals, the desired parameter or set of parameters is computed, obtaining a value for each time interval. For example, computing the 95th percentile of the range would lead to the array shown in Equation (8):

(8) $$E_{P95(range)}=[E_{P95\left(range\right)}^{\Delta t_{1}}\;E_{P95\left(range\right)}^{\Delta t_{2}}\;\ldots \;\;E_{P95\left(range\right)}^{\Delta t_{N}}]$$

**Figure 8:**
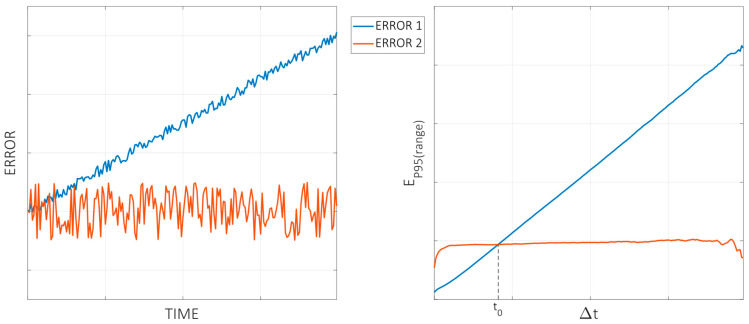
Illustrative example of two thermal distortion curves evaluated according to the procedure described in this work. (**Left**) Two different thermal distortion curves are shown versus time. (**Right**) The parameter $E_{P95(range)}$ for both error curves versus the corresponding time interval.

The first value of the array provides information about the thermal behavior in the resolution limit of the test, dt, as the last value is directly the range of the thermal error over the full duration of the test. To better illustrate the usefulness of this procedure to evaluate thermal results, a naive example is shown in the following with synthetically generated data. The left part of Figure 8 shows two thermal distortion curves with clear, distinct behaviors. The blue line (“error 1”) shows a drift that extends over the whole duration of the test. The red line (“error 2”) shows a stable long-term behavior, but a bigger noise-type error compared to the blue line. Assuming a similar measurement uncertainty, the $E_{P95(range)}$ parameter is computed for each curve and is shown in the right part of Figure 8 in order to compare both behaviors.

The red and blue lines on the right-hand side show the $E_{P95(range)}$ parameter for both cases, which can be interpreted as follows: “when working at operation intervals of length $\Delta t_{n}$ it has been measured that the thermal error variation is below the value $E_{P95\left(range\right)}^{\Delta t_{n}}$ in the 95% of the cases”. In other words, when performing machining operations of a duration $\Delta t_{n}$, starting at any arbitrary moment, the expectation is that the thermal error will vary less than that in the 95% of the cases. According to this, the graphs show that, when working in time intervals below $\Delta t_{n}=t_{0},$ the behavior represented by the blue line seems to be a better option, and when working in longer time intervals, the red one should provide a better accuracy. The selection of one over the other will depend on the specific needs of the machine user.

### 2.4. Validation on Virtual Machining Tests According to ISO—10791

**Figure 9:**
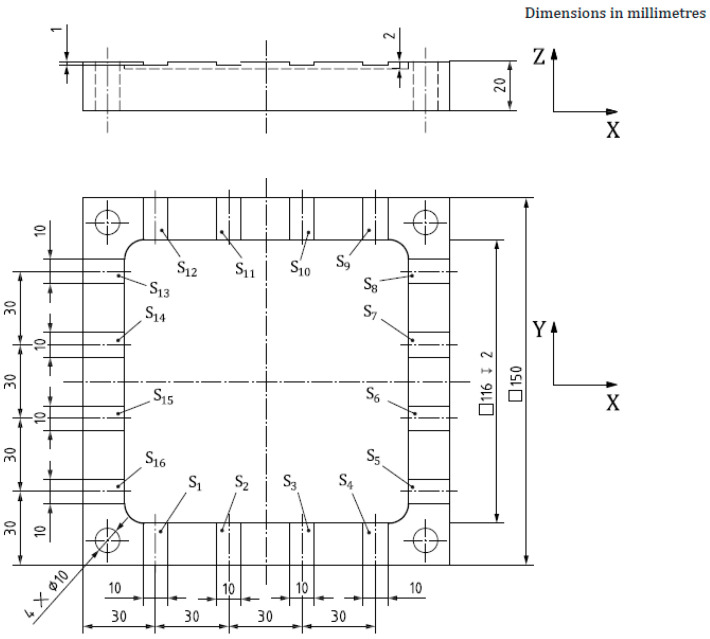
Figure taken from [33] showing the workpiece from the test to evaluate machine tool distortion in the tool direction described in the standard ISO 10791 Part 10, in Section A.3.

An additional virtual machining test is proposed to further validate the results. This test simulates the trajectories of machining tests explained in Annex A of the ISO 10791—Part 10 standard. The test is proposed in Section A.3 under the name “Machining test to evaluate the machine tool distortion in the axial direction of the tool”, which is the Z direction for this machine. The test consists of machining 16 slots (S1 to S16) on a square-shaped workpiece, evenly spaced in time. The first slot (S1) is used as a reference and the relative axial error is reported for the remaining 15 slots. According to the standard, the waiting time between machining the slots can be modified depending on the time it takes thermal deformations to occur. For this case, a waiting time of 1 h is selected, resulting in a 15 h interval between the first and last slot. The finished geometry can be seen in Figure 9 [33]. These virtual tests will be simulated starting at different times of the available validation test so that the digital twin’s compensation behavior can be evaluated under different conditions.

## 3. Results

**Figure 10:**
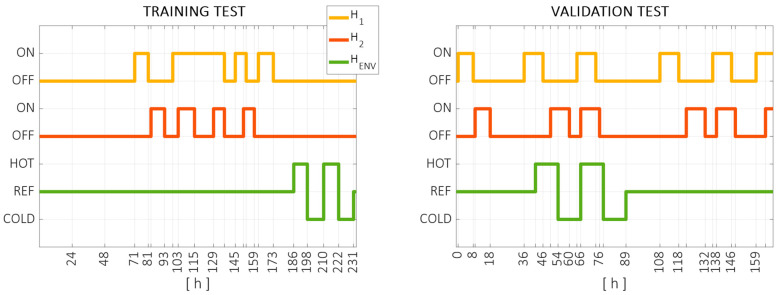
Thermal conditions applied to the training test (**left**) and the validation test (**right**). The local heat sources H_1_ (yellow) and H_2_ (red) are switched on and off during the test, as the ambient (green) is forced to cool down, maintain, or heat up the workshop temperature. Vertical marks are added in switching moments.

Following the measurement procedure defined in Section 2.1, continuous measurements were performed on the machine over two different time spans. The training test was carried out from 24 March to 3 April, lasting 234 h (approximately 10 days). The validation test (testing data set) was carried out from 8 June 8 to 15 June 15, lasting 169 h (approximately 7 days). Different thermal conditions were induced on the machine, using the three controlled heat sources defined in Figure 3. The thermal conditions applied throughout the training and validation tests can be seen in Figure 10.

**Figure 11:**
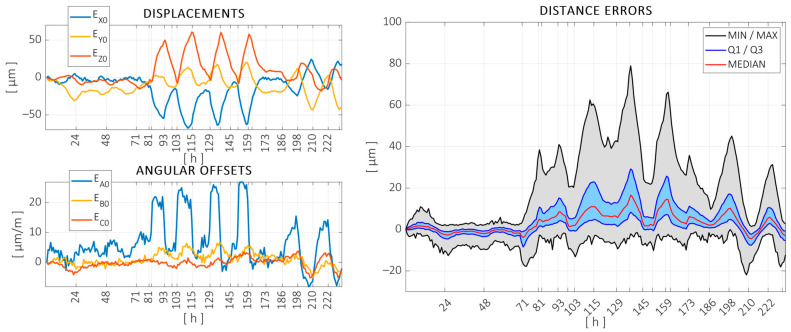
Measured offset errors (**left**) and distance errors measured on the artifact (**right**) throughout the 234 h long calibration test.

The results obtained in the training and validation tests are summarized in the following. Figure 11 shows the displacements measured in the training test. The left-hand side of the figure shows the position and orientation changes in the volume as a whole, as defined in [58]. The measured position deviations are expressed in the same coordinate system as the machine, and vertical grid markers were added at time steps where the local heat sources were switched on/off or when the ambient environment was forced to heat up or cool down. The right-hand side of the figure shows the variation in the distances measured between spheres. Since a large number of distances were measured at each measurement cycle, the distribution of the variation in these distances is represented through the following quartiles: minimum, maximum, median, and first (Q1) and third (Q3) quartiles. The variations in these measured distances were in the same order of magnitude as in the fixed balls, showing that both the overall drift and the volumetric variation are relevant.

**Figure 12:**
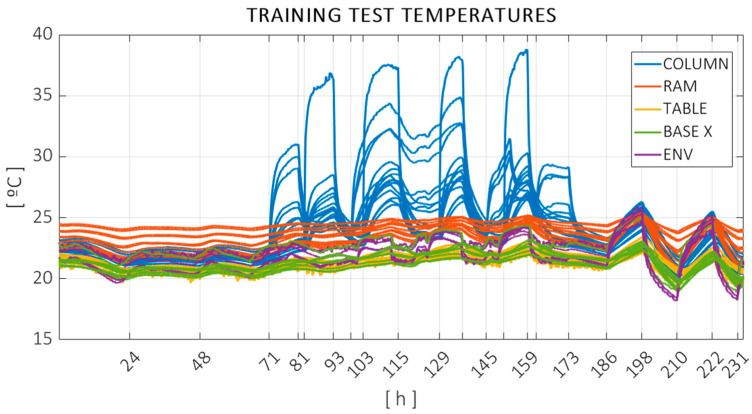
Temperatures recorded during the training test, color coded according to machine components: column (blue), ram (red), X base (green), workpiece table (yellow), and ambient probes (purple).

The measured temperatures can be observed in Figure 12, color coded according to Figure 4. The same vertical markers are added to indicate heat sources switching on or off. Temperature data were acquired every 30 s.

As can be seen in Figure 11 and Figure 12, the first 70 h of the test, under just the unforced ambient variation, were relatively stable, with little consequent variation in the error. The central part of the test, where the localized heat sources were activated, was noted primarily in the column temperatures as expected, with considerable effects on both the offset and distance errors. The last 48 h of the test were performed under forced ambient temperature changes. The evolution of the temperatures was highly correlated in this section, with more than 7 °C variation measured with the ambient probes and more mitigated changes in the structure. The variation in the volumetric error was also significant in this period.

**Figure 13:**
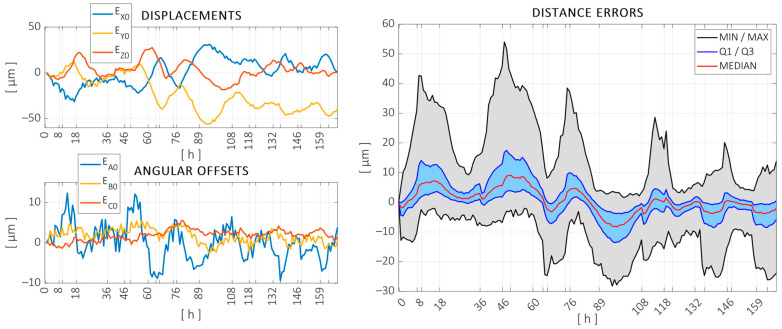
Measured offset errors (**left**) and distance errors measured on the artifact (**right**) throughout the 169 h long validation test.

**Figure 14:**
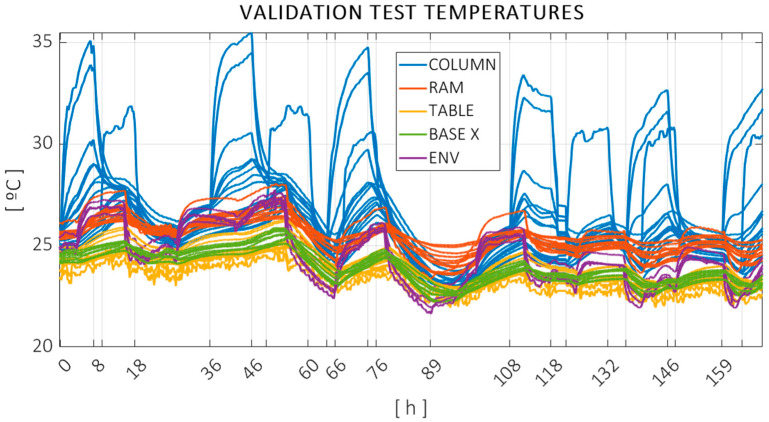
Temperatures recorded during the validation test, color coded according to machine components: column (blue), ram (red), X base (green), workpiece table (yellow), and ambient probes (purple).

Similarly, Figure 13 and Figure 14 show the measured errors and the temperature evolution throughout the validation test.

Comparing Figure 12 and Figure 14, it can be observed that the mean temperature of the validation test was higher than that of the training test. The average ambient temperature increased from 22 °C to 24.7 °C from training to validation. Furthermore, the changes between daytime and nighttime were bigger and more sudden, as the climate of the workshop was active during the day and switched off from 6 p.m. on. The validation tests’ ambient temperature increased to 27 °C during some nights. This shows the seasonal behavior of thermal issues and entails an additional difficulty when trying to predict the thermal errors of a machine tool.

## 4. Discussion

### 4.1. Identification of the Digital Twin

**Figure 15:**
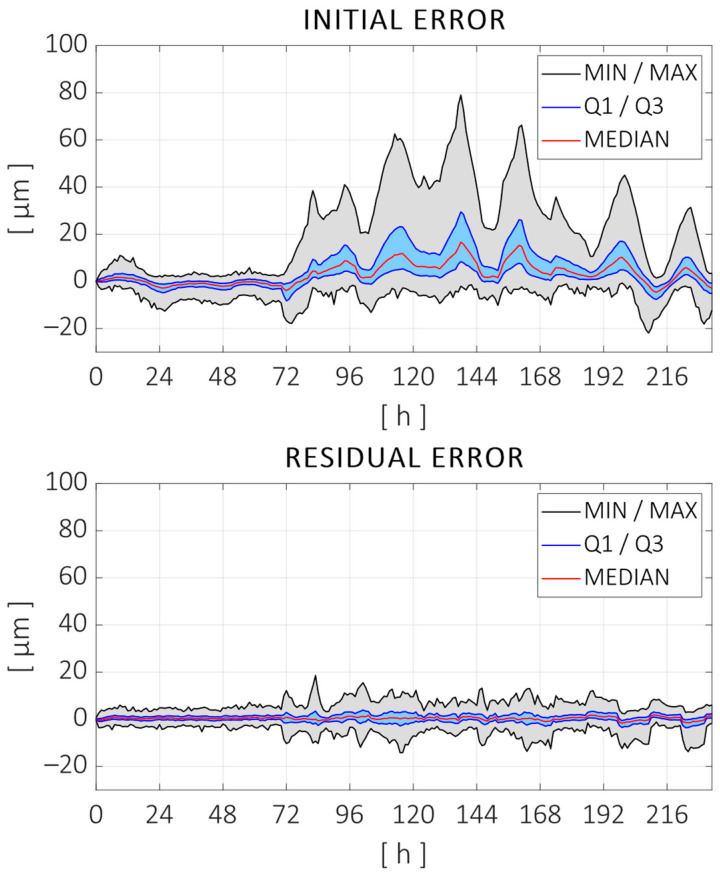
(**Top**) Measured distance errors during the training test. (**Bottom**) Residual error after adjusting thermal regression models for each error parameter.

Following the procedure shown in Figure 5 for the identification of a digital twin for volumetric thermal error prediction, volumetric error estimation was performed with the distances measured at each time step in the training test. Therefore, the evolutions of each of the parameters defining the component errors were obtained for 234 h. The regression model described in Section 2.2 was estimated for each of the parameters, which was used along with the machine model M to compare the errors during the training test. The initial distance errors are compared to those that would be measured if the digital twin was used to compensate for the position of the machine during the training test in Figure 15.

**Figure 16:**
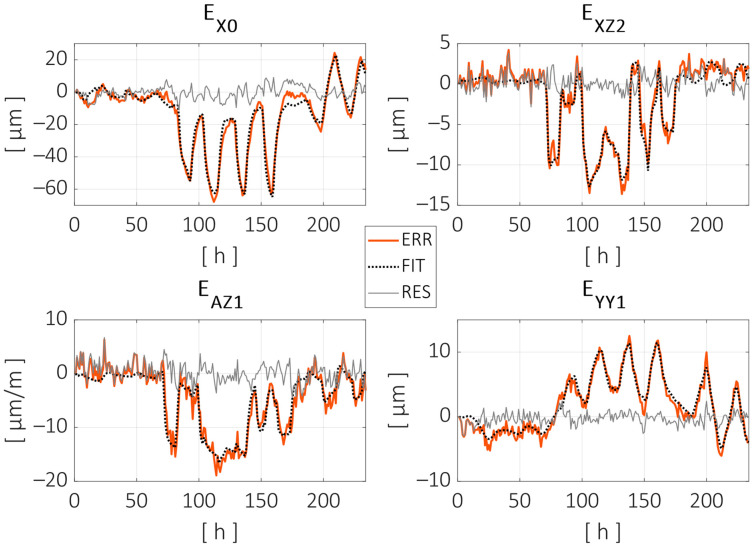
Parameter evolution and model fitting for some of the component error parameters. Measured parameter evolution is pictured with a red line, the thermal model fitting with a dotted black line and the residual error after fitting with a grey line.

As can be observed, the thermal regression model adjusted most of the distance errors measured during the test. The peak-to-peak error was reduced from 101 µm to 33 µm (68% reduction), and the root mean square (RMS) error was reduced from 9.7 µm to 2.5 µm (74% reduction). Figure 16 shows the fitting curves adjusted for some of the error parameters. Displacement parameters (positioning and straightness) are expressed in microns (µm), and the angular errors are expressed in microns per meter (µm/m), assuming small-angle approximation. The parameter names are given according to the ISO—230 Part 1 standard, with an additional numerical term at the end indicating the order of the parameters for the specific component error. In this case, the following parameters are shown: the zero-order error in the X direction, the second-order parameter of the Z axis straightness in the X direction, the first-order (linear) parameter of the Z axis angular error around the X axis, and the first-order parameter of the positioning error of the Y axis (linear expansion).

Each of the parameters shown in Figure 16 were fitted using the ARX model described in Equation (4). A summary of all geometric error parameters can be found in Table 1. This includes the initial peak-to-peak and RMS values, the improvements after the thermal model training, the number of temperature inputs, and the inclusion or exclusion of the autoregression parameter $a_{m}$.

As can be seen in Table 1, the number of temperature inputs varied from two to five for different error parameters, a number that was chosen by comparing different models using the information criteria described in Section 2.2. Using the same criteria, the autoregressive parameter ($a_{m}$) was included in only 2 of the 24 parameters, i.e., the model selection criteria established that, in most cases, there was no significant improvement with adding the autoregressive parameter or priority was given to adding extra temperature inputs (e.g., using two temperature inputs rather than one temperature plus the autoregressive parameter). Even in the two cases where the parameter $a_{m}$ was used, the improvement was not especially significant, with differences in the RMS reduction of less than 5%.

Therefore, the estimation models of most of the parameters resulted in simple Multiple Linear Regression models rather than ARX models. Each parameter was predicted by a linear combination of certain temperature values at a specific time instant. This reinforces the underlying idea that the relation between temperatures and deformations can be established by such direct models if enough information on the temperature field is available.

The interpretation of the results, i.e., how different heat sources affect the machine component errors or tool trajectories, can be easily performed using the machine model $M_{T}(X,E(T))$, which, once it is trained, can simulate any machine position or trajectory. A comprehensive analysis of the thermal volumetric errors using this approach can be found in [58].

### 4.2. Validation and Error Prediction of the Digital Twin

**Figure 17:**
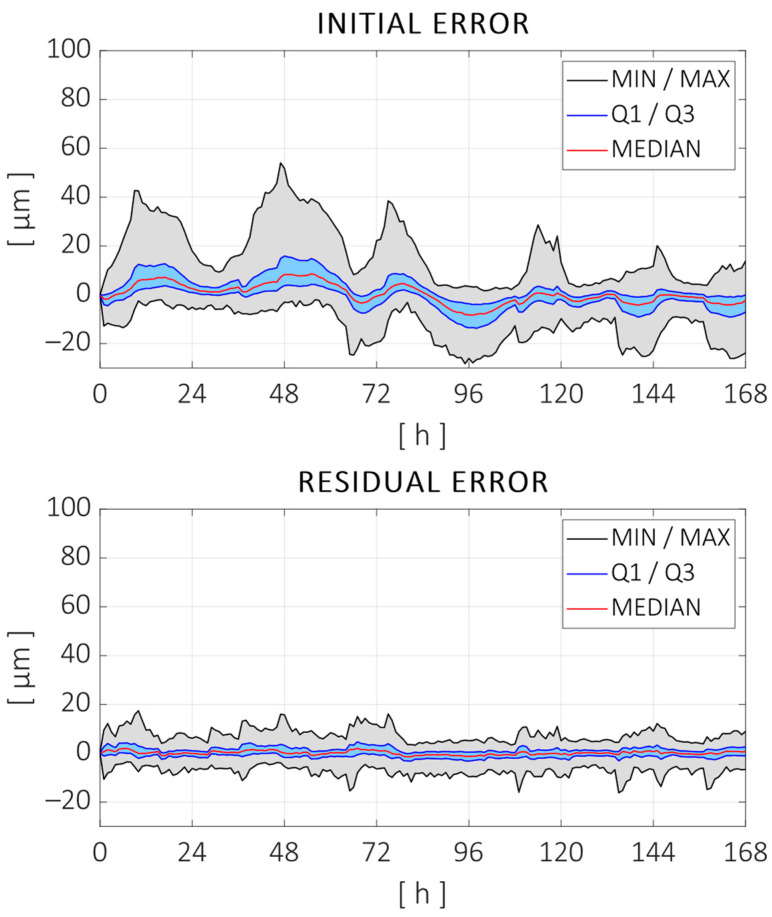
(**Top**) Measured distance errors during the validation test. (**Bottom**) Residual error after compensating the errors with the regression model obtained with the training test.

The use of the regression model obtained in the previous section as a digital twin for the volumetric thermal error compensation is validated using the 168 h long validation test described in Section 3. The ball position and distance variations measured in the validation test are compared to those estimated using the regression model obtained with the training test and the temperature inputs recorded during the validation test. The kinematic machine model $M_{T}$ is used to calculate the estimation of distances from the individual error parameters. Figure 17 shows the measured distance errors and the residuals after compensating with the estimated ones.

As can be observed, the thermal regression model compensated for most of the distance errors measured during the validation test. The peak-to-peak error was reduced from 82 µm to 33 µm (59% reduction), and the root mean square (RMS) error was reduced from 7.5 µm to 2.9 µm (61% reduction). As expected, the improvement in the testing data was worse than the fitting quality with the training data. Still, the reduction rates were close enough and the compensation model is considered to be sufficiently robust considering the following:

- The training and testing datasets are of a similar size (58%/42% split), while the usual rates are around 80%/20%.
- There is almost a three-month difference between both tests, which implies seasonal differences in climate and possible changes in the machine state as it continued its normal operation in between.
- The thermal load cycle changed, and the heat sources are recombined in a totally different sequence with respect to the training test.

**Figure 18:**
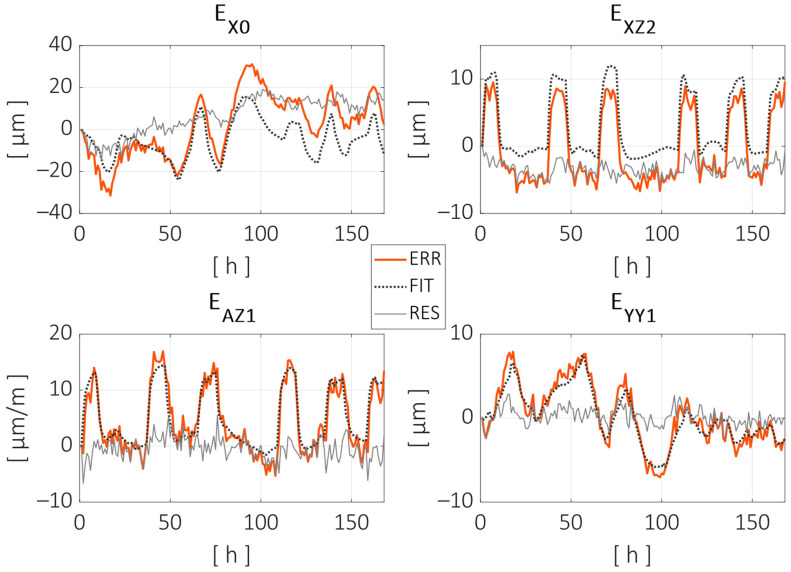
Parameter evolution and model estimation for some of the component error parameters during the validation test. Measured parameter evolution is pictured with a red line, the thermal model fitting with a dotted black line and the residual error after fitting with a grey line.

Figure 18 shows the evolution of the individual error parameters, similarly to Figure 16. This time, the fitting curves are estimations generated by the regression model according to the temperature inputs during the validation test.

Figure 18 shows some of the limitations and strengths of the compensation model. As can be seen with the parameters $E_{X0}$ and $E_{XZ2}$, the compensation model predicts the short-term variations due to changes in the local heat conditions relatively well, but there is a long-term drift in the difference between the measured and compensated values, especially in the $E_{X0}$ case. A long-term defect in the compensation model is generally considered to be less critical, as a typical machining process usually lasts several hours at most, and the workpiece can be referenced at the beginning of the process or between different operations.

### 4.3. Analysis of the Volumetric Error Compensation

For a general analysis of the volumetric error of the machine, three straight trajectories are simulated, with each one aligned with a machine axis and all crossing each other at the center of the measured working volume.

**Figure 19:**
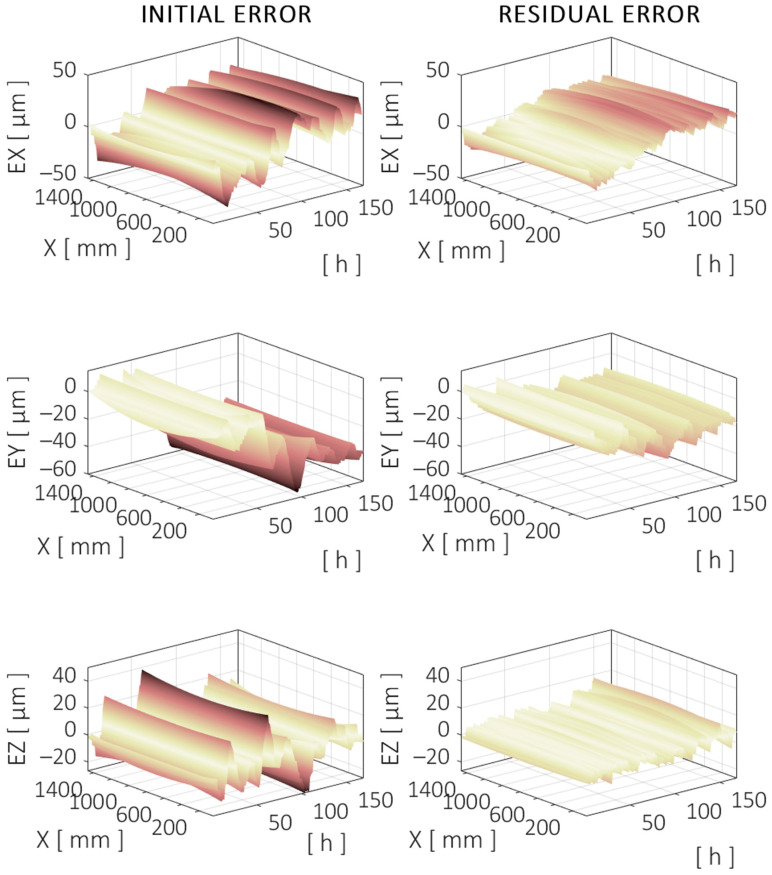
Evolution of the directional errors at TCP over a centered trajectory along the X axis during the validation test. Initial (**left**) and compensated (**right**) errors are shown. Darker color means higher (absolute) error. Same color scale applies to all figures.

The trajectory along the X axis corresponds to the linear motion at the midrange of the Y and Z axes (X = [30–1460] mm, Z = 975 mm, and Y = 625 mm). The TCP component errors are shown in Figure 19, which correspond to the positioning (X) and straightness (Z and Y) errors of the X axis along the specified trajectory. The evolution of the error is shown during the 7-day validation test. The machine model *M* is used to simulate the trajectories and the errors of the machine.

**Figure 20:**
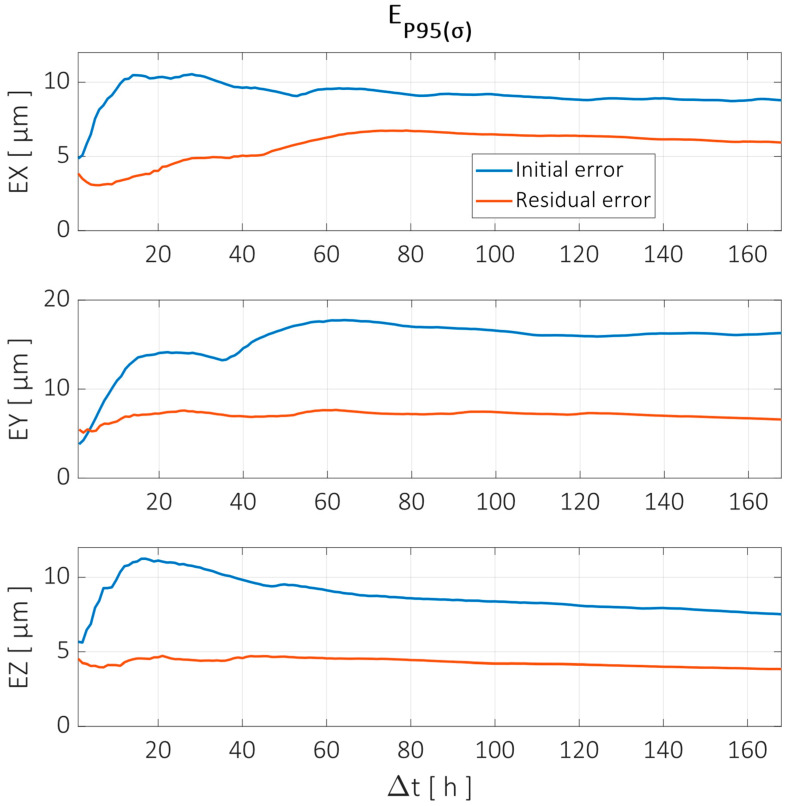
Error reduction for X axis trajectory evaluated at different time intervals according to the parameter $E_{P95(\sigma )}$. Error components in X, Y, and Z directions are shown before (blue line) and after compensation (red line).

To evaluate the error reduction achieved by the compensation model, the methodology defined in Section 2.3 is used. The parameter selected to evaluate the error is the standard deviation of each time interval, i.e., the dispersion of the error relative to its mean in the specific interval $\Delta t_{n}$. The value representing each $\Delta t_{n}$ corresponds to the 95th percentile of all the intervals evaluated, referred to as $E_{P95(\sigma )}$. Figure 20 shows the reduction achieved for different $\Delta t_{n}$ for each component error in the X, Y, and Z directions for the X axis trajectory shown in Figure 19.

Figure 20 shows the different behaviors for the residual errors in each direction. When looking for the total duration of the test (Δt=168 h), the overall reduction achieved in the X direction was only 33%. However, there were time intervals where the compensation model performed much better. For example, the time intervals between 5 h and 20 h showed a reduction of 60% or greater when comparing the initial and residual errors after compensation. This effect is clearly observed in the top two graphs from Figure 19, where a long-term drift is still noticeable after compensation, but the dominant shorter-term ups and downs are greatly reduced. This may suggest that the strength of this compensation model in the X direction is in predicting (relatively) short-term behavior rather than assuring long-term stability.

Regarding the Y direction, the performance of the compensation model was quite stable for every time interval when looking at the absolute values of the residual error. The original error, on the other hand, was already quite low (<4 µm) when looking at 1 h and 2 h intervals, and the compensation model slightly worsened the original results. However, for longer time intervals, reductions up to 60% were achieved.

The Z direction error showed the greatest variability in the time intervals between 10 h and 30 h, as shorter and longer intervals showed smaller values. The compensation model performance was quite stable within the whole range, with a reduction of up to 62% in the mentioned interval and a 50% reduction when looking at the full duration of the test.

**Figure 21:**
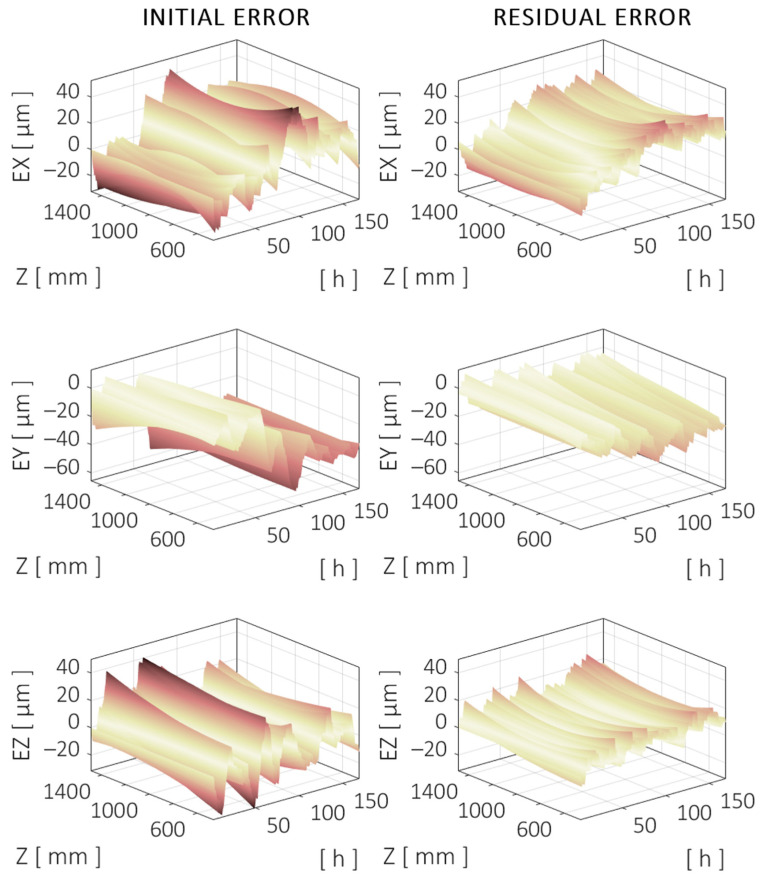
Evolution of the directional errors at TCP over a centered trajectory along the Y axis during the validation test. Initial (**left**) and compensated (**right**) errors are shown.

The trajectory along the Z axis corresponds to the linear motion at the midrange of the X and Y axes (Z = [460–1490] mm, X = 745 mm, and Y = 625 mm). The TCP component errors are shown in Figure 21, which correspond to the positioning (Z) and straightness (X and Y) errors of the Z axis along the specified trajectory.

**Figure 22:**
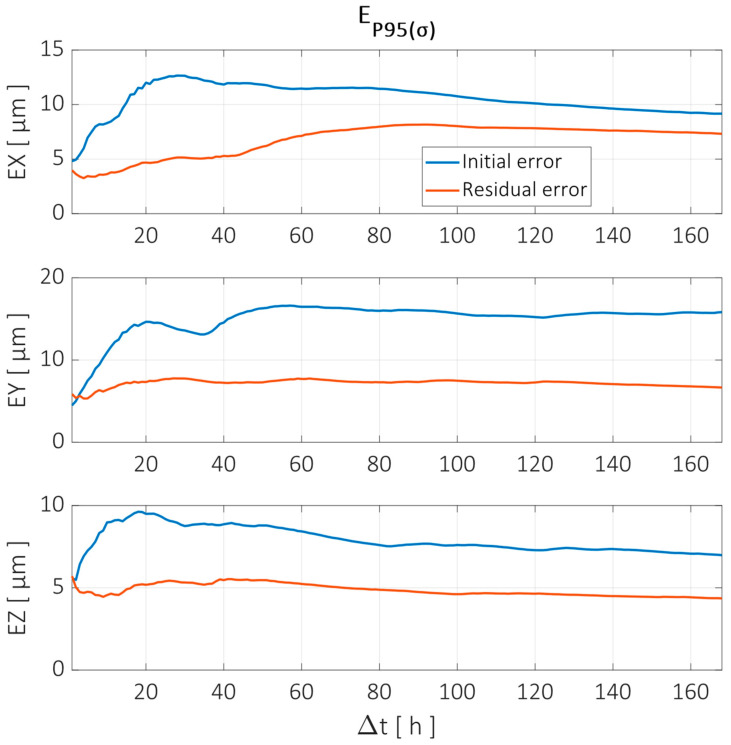
Error reduction for Z axis trajectory evaluated at different time intervals according to the parameter $E_{P95(\sigma )}$. Error components in X, Y, and Z directions are shown before (blue line) and after compensation (red line).

Similarly, the errors before and after compensation are evaluated for different time intervals. This is shown in Figure 22.

The error reduction achieved in the Y trajectory by the compensation model showed similar behavior to that in the X trajectory. The X direction error was reduced by more than 50% in the time intervals between 20 h and 40 h, but the long-term reduction was less than 20%. The error compensation showed better behavior in the other directions, with better long-term results for the Y error, while the Z error reduction was better in the 10–20 h intervals.

**Figure 23:**
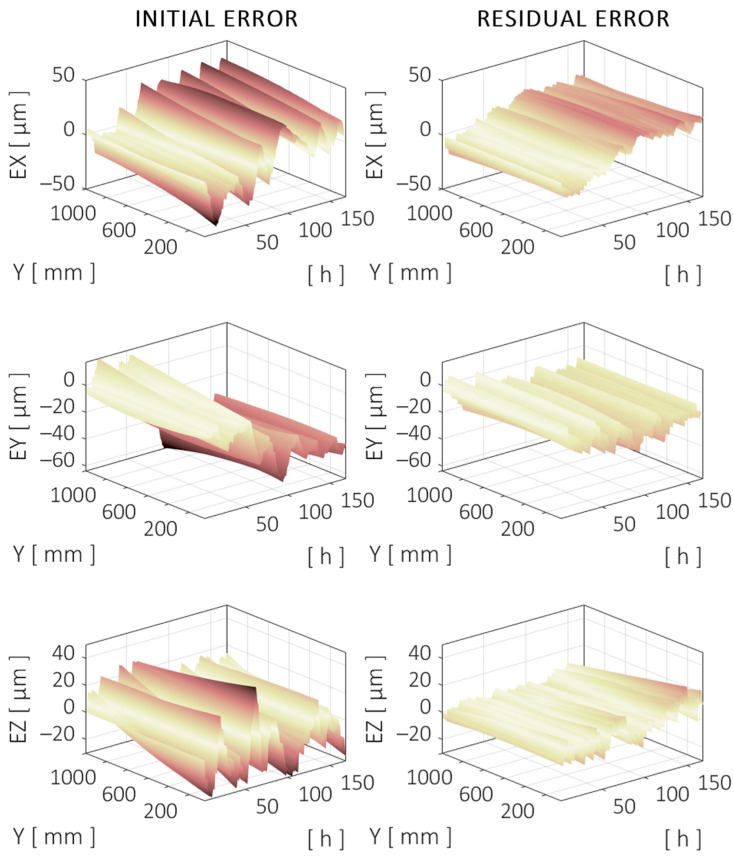
Evolution of the directional errors at TCP over a centered trajectory along the Z axis during the validation test. Initial (**left**) and compensated (**right**) errors are shown.

The trajectory along the Y axis corresponds to the linear motion at the midrange of the X and Z axes (Y = [50–1200] mm, X = 745 mm, and Z = 975 mm). The TCP component errors are shown in Figure 23, which correspond to the positioning (Y) and straightness (X and Z) errors of the Y axis along the specified trajectory.

**Figure 24:**
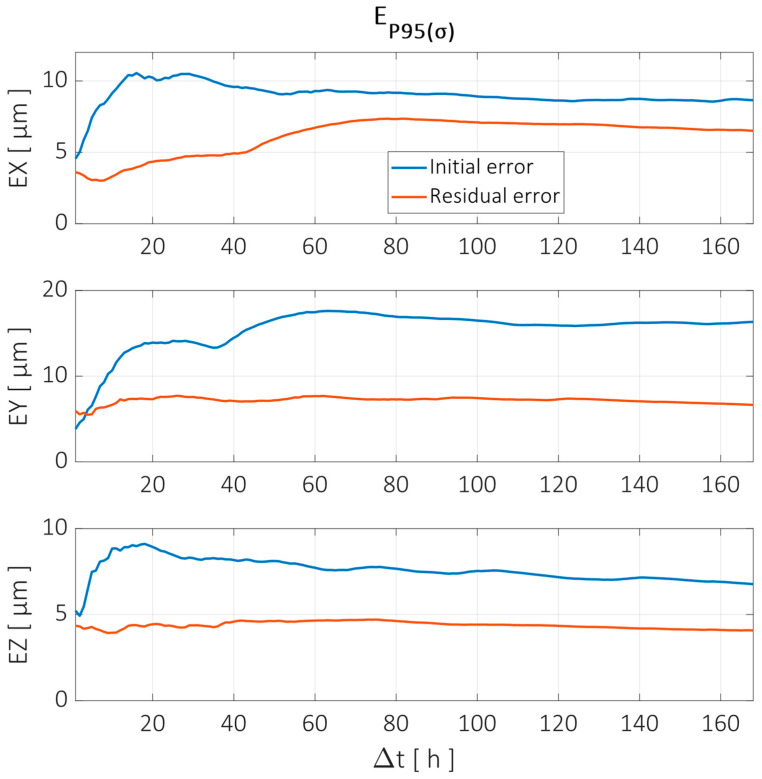
Error reduction for Y axis trajectory evaluated at different time intervals according to the parameter $E_{P95(\sigma )}$. Error components in X, Y, and Z directions are shown before (blue line) and after compensation (red line).

The error analysis in the time interval domain showed similar behavior to the X and Z trajectories, as can be seen in Figure 24.

### 4.4. Validation on Virtual Machining Tests According to ISO—10791

As seen in the previous section, the digital twin obtained above $M_{T}(X,E(T))$ can simulate any trajectory under various thermal conditions and make a prediction of the errors at the TCP. To further validate the results of the compensation model, the virtual test presented in Section 2.4 is carried out.

**Figure 25:**
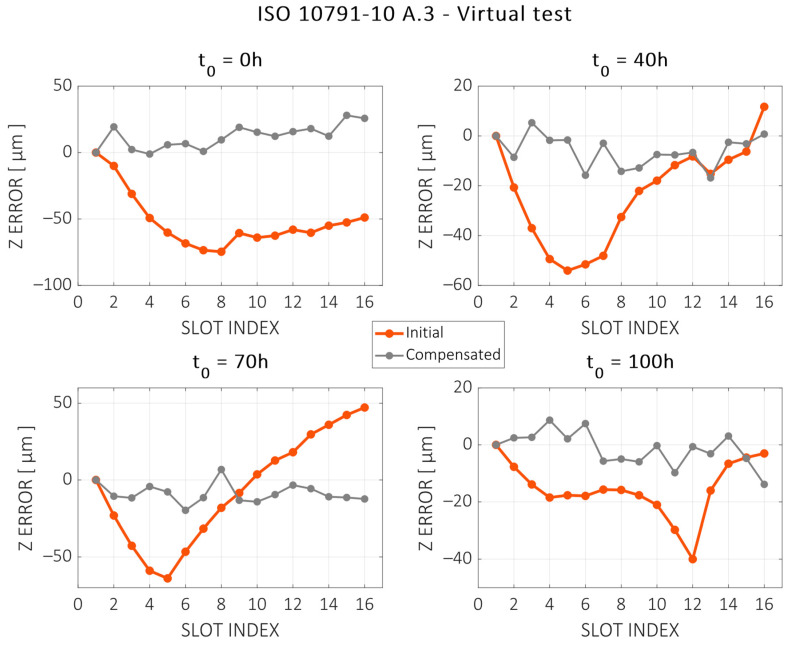
Virtual machining test results, presented according to standard ISO—10791 Part 10. Initial (red) and compensated (grey) errors are shown with the machining test simulated at different intervals of the validation test.

The machining trajectories are simulated using the digital twin of the machine $M_{T}$ on different time intervals over the duration of the test. Figure 25 shows the evaluation of thermal distortions in the Z direction for machining tests starting at the 0 h, 40 h, 70 h, and 100 h marks of the validation test (see Section 3). Table 2 shows the error reduction by the means of range (peak-to-peak error) and RMS for these four cases.

**Figure 26:**
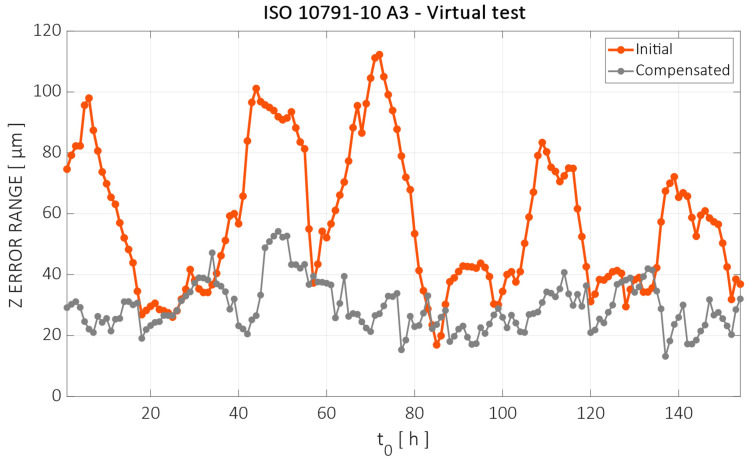
The error range values for the 15 h long A.3 machining test starting at successive time steps before and after compensation.

In order to generalize the results throughout all the validation tests, the evaluation method pictured in Figure 7 is used. A moving window of 15 h is used and the test is simulated starting at each time step, with the first one being the 0–15 h interval and the last one ranging from 153 h to 168 h. Figure 26 shows the error range (peak-to-peak value) before and after compensation for the tests starting at successive time steps.

The 15 h interval error variation was reduced from 57.5 µm to 29.5 µm, on average, a 49% reduction. The maximum error was reduced from 112 µm to 54 µm, a 52% reduction. In general, the greatest reductions were achieved in intervals where the error variation was high (e.g., tests starting 60 h and 80 h), as there was almost no improvement in intervals of a lower variation (e.g., between 20 h and 30 h).

### 4.5. Modeling Considerations and Uncertainties

The results shown throughout Section 4 show a significant reduction in the thermal error after compensation was applied with the digital twin of the machine. However, the error prediction was far from perfect and showed some limitations. This section analyzes the modeling assumptions and uncertainty sources that may have been the causes of these limitations. The digital twin for volumetric thermal error compensation was built upon a double assumption, as follows:

- The first was that geometric errors can be approximated by lower-order polynomials of smooth forms. In principle, these geometric errors can adopt any arbitrary form, but it can be useful to approximate them by the means of different polynomial approximations. This simplifies the characterization problem at the expense of losing some, hopefully residual, geometric information.
- The second and less common assumption was that the parameters related to the polynomials characterizing the geometric errors experience a temporal variation that can be predicted by the means of temperature variations or other related inputs. In other words, not only can geometric errors be approximated by polynomials, but their variation due to temperature changes can also be approximated. Furthermore, these changes can be predicted by measuring thermal inputs. The existence of such relations between inputs and specific parameter variations is not granted, and it was the key aspect in establishing a two-step thermal volumetric compensation model that extends from temperatures to parameters and from parameters to geometric error values at any given position and time.

The first assumption was expected to be fulfilled, as this approximation by polynomials is common knowledge in the field of the geometric error characterization of machine tools, and was demonstrated in the work preceding this publication [58]. The compensation results shown in Section 4 demonstrated that the second assumption turned out to be appropriate, at least partially. A great part of the thermal volumetric error variation was predicted and compensated, and a significantly good correlation was found between temperatures and most of the parameters. The deviations and improvement rates shown by the results in the validation sections are considered to be robust and reliable, as a comprehensive validation procedure was carried out, with a duration comparable to the training test, which included seasonal variations and pattern changes in thermal load sequences.

The kinematic model of the machine (denoted as M in this work) was the key enabler in order to understand and validate the measurements and compensation results. The model incorporated the position-dependent behavior of geometric errors, which is a usual functionality of this kind of model, but this time with the addition of temperature-dependent effects. In other words, using the machine position and temperatures as inputs, the model predicts the error at the TCP in any point in space. This model was a powerful tool to validate our results by the means of different trajectories and workpiece machining tests. Furthermore, the model could be used to simulate future thermal scenarios by synthetically generating temperature data and using them as inputs to simulate different trajectories.

Regarding the model established between temperatures and thermal distortions, it should be noted that the model selection based on the information criteria discarded ARX models and favored simple Multiple Linear Regression, which established direct relations between multiple temperature inputs and thermal distortions. Considering the fact that the relation between a body’s temperature and thermal distortion is immediate, it seems logical that, in the hypothetical case where the full temperature field is known, a direct model (i.e., MLR model) should be identified between both. However, when measuring one or few temperature spots, it is usual to observe delays and “dumped” thermal effects between them and the distortion measured on a single point, suggesting that models incorporating such effects (such as ARX models or transfer functions) are more appropriate for these cases. Therefore, it seems reasonable to think that, at a certain point between measuring one or few temperature spots and knowing the full temperature field, MLR models will begin to become more and more suitable, to the detriment of other types of models. This seems to be the case for the thermal effects shown in this work.

However, the results after compensation were far from perfect, as can be seen in the validation procedures shown in Section 4. The results were dissimilar when compensating for the error changes of different time intervals, and small but significant long-term drifts remained even after compensation. The main aspects and uncertainty sources responsible of this suboptimal performance are listed in the following:

- Uncertainties related to the measurement system and the calibration procedure. The details of these were extensively discussed in works preceding this publication [57,58] and included aspects such as sensor uncertainty, machine repeatability, ball sphericity, and especially the time taken by the calibration procedure to complete a full measurement cycle, as thermal effects continuously change.
- To what extent are the two assumptions fulfilled? The assumptions listed earlier in this section may not be a good enough approximation of the actual physical phenomena, i.e., the polynomial approximation may not be appropriate to model the actual values of geometric errors, and their variation may not be predictable with the measured inputs, at least to a certain extent.
- Incomplete information of the temperature field. The fact that the thermal distortion of the body can be predicted by knowing its temperature is only true if the full temperature field is measured. Predicting such distortions by measuring only some specific spots (as is the case) is only an approximation that may work to a certain extent. This is especially true when working with big machines under environmental thermal effects that are distributed over large areas. The limitation of only measuring 50 spots instead of the full temperature field of the machine could result in thermal effects not being measured at all or without the necessary accuracy.

## 5. Conclusions

In this work, a novel method to measure and compensate for the thermal variations in volumetric errors was presented. The compensation was performed with a digital twin that can be implemented in the control of machines, continuously estimating the positioning error as a function of some measured temperatures and the axes’ positions. In contrast with the typical approach to separating geometric and thermal errors, this work considered that all geometric errors present in a machine tool can, and ultimately do, change with the influence of the temperature over time. Therefore, a unified methodology was presented, combining the spatial (volumetric) dimension to characterize the geometric errors and the temporal dimension needed to measure thermal effects. This was achieved by implementing a fully automated measuring methodology to calibrate geometric errors, which was then repeated over time.

The improvement in the error after compensation was significant but limited due to the several factors and modeling limitations discussed in Section 4.5. However, the results are considered to be robust enough to represent a promising approach, as an extensive validation test was performed, varying the conditions of the calibration test.

Finally, in Section 2.3, a novel systematic method to evaluate thermal behavior was proposed. Roughly inspired by the frequency domain analysis of time series, this method allows for selecting the more appropriate parameter or evaluation metric and applying it to increasing time intervals of the corresponding signal (e.g., thermal distortion measured at one point). This results in a graphical representation loosely mirroring a frequency response function, which permits a quantitative and qualitative evaluation of the errors at different time intervals at a glance. This method permits simple and visual comparisons between the measured error and the residual after compensation is applied, discerning, for example, short-term and long-term compensation capabilities, and overcoming simplistic numerical representations by just reporting overall results. This methodology can be used not only to evaluate compensation models, but also to compare the thermal behaviors of different machines or situations and selecting the optimal time intervals for machining.

## Figures and Tables

**Table 1:** Summary of estimated parameters with the training data.

Parameter	P2P_0_ [µm]	P2P_FINAL_ [%]	RMS_0_ [µm]	RMS_FINAL_ [%]	No. of Inputs	*a_m_* (Yes/No)
$E_{X0}$	92.2	78.1	24.9	84.7	3	0
$E_{Z0}$	78.7	72.4	21.2	79.4	2	0
$E_{Y0}$	63.9	64.8	16.9	71	5	1
$E_{A0}$	35.1	50.0	10.4	72.2	3	0
$E_{C0}$	8.9	44.4	1.7	42.6	2	0
$E_{B0}$	11.9	56.8	2.5	59.6	2	0
$E_{XX1}$	23.6	63.3	4.6	67.5	3	0
$E_{XX2}$	9.2	8.1	2	21.8	2	0
$E_{ZX2}$	23.7	48.9	8.2	71.3	4	0
$E_{AX2}$	19.8	61.5	7.8	79.3	3	0
$E_{BX1}$	29.9	55.3	6.8	60.6	2	1
$E_{ZZ1}$	40.9	51.1	8.2	60.9	4	0
$E_{ZZ2}$	11.1	56.0	3.1	68.4	3	0
$E_{XZ1}$	19	2.2	3.9	12.9	2	0
$E_{XZ2}$	17.8	62.2	5	78.8	4	0
$E_{YZ2}$	6.2	39.9	2	68.3	4	0
$E_{CZ1}$	9.2	7.3	2.2	38.9	4	0
$E_{AZ1}$	25.2	59.2	7.5	77.4	5	0
$E_{YY1}$	18.6	74.3	4.7	81.7	5	0
$E_{YY2}$	7.7	52.6	2.1	68.7	2	0
$E_{ZY1}$	19.5	61.3	7	80.4	5	0
$E_{ZY2}$	7.6	34.0	1.7	50.9	4	0
$E_{XY1}$	33.7	75.9	12.3	87.4	4	0
$E_{XY2}$	5.8	13.1	1.3	21.1	2	0

**Table 2:** Error reduction for the ISO 10791-10 A.3 machining test for different intervals of the validation test.

$t_{0}$	P2P_0_ [µm]	P2P_FINAL_ [µm]	P2P_RED_ [%]	RMS_0_ [µm]	RMS_0_ [µm]	RMS_FINAL_ [%]
0 h	74.6	29.1	61.0	55.7	14.7	73.6
40 h	65.8	22.1	66.3	30.3	8.6	71.6
70 h	111.2	26.6	76.1	35.5	10.6	70.0
100 h	40.0	22.5	43.8	18.2	6.0	67.2

## Data Availability

All data supporting the findings of this study are available within the paper. Raw data are available from the corresponding author upon reasonable request.
